# Disrupted sensory interhemispheric synchronization in schizophrenia: a frequency-resolved VMHC analysis

**DOI:** 10.3389/fpsyt.2026.1833948

**Published:** 2026-06-12

**Authors:** Lei Peng, Huiyun He, Zhi Huang, Yongshan Wang

**Affiliations:** The Clinical Hospital of Chengdu Brain Science Institute, Ministry of Education (MOE) Key Lab for Neuroinformation, University of Electronic Science and Technology of China, Chengdu, China

**Keywords:** fMRI, frequency-band, primary network, schizophrenia, voxel-mirrored homotopic connectivity

## Abstract

**Background:**

Aberrant interhemispheric functional connectivity has been implicated in the pathophysiology of schizophrenia. Voxel-mirrored homotopic connectivity (VMHC) provides a reliable measure of interhemispheric synchronization, yet the frequency-specific characteristics of VMHC alterations in schizophrenia remain poorly understood.

**Methods:**

Resting-state functional magnetic resonance imaging data were analyzed in patients with schizophrenia and matched healthy controls. VMHC was computed across frequency bands, with particular focus on slow-4 and slow-5 oscillations. Group differences, as well as group-by-frequency interactions, were assessed to identify frequency-specific disruption.

**Result:**

Patients with schizophrenia exhibited significantly reduced VMHC within key sensory networks, including primary visual and sensorimotor regions. Frequency-specific analyses revealed higher VMHC at slow-5 compared with slow-4 in visual gyrus and subcortical regions. Significant group-by-frequency interaction effects were observed in the middle occipital gyrus and postcentral gyrus, with *post-hoc* analyses indicating selectively reduced slow-4 VMHC in patients with schizophrenia.

**Conclusion:**

This study demonstrates frequency-dependent reductions of interhemispheric connectivity in schizophrenia, particularly within sensory systems. The findings highlight the disrupted integration of primary perceptual and motor-related processes as a core feature of schizophrenia and emphasize the utility of frequency-resolved VMHC analyses for refining our understanding of network dysfunction. Future longitudinal studies are warranted to determine the clinical significance of these frequency-specific alterations in illness progression and treatment response.

## 1 Introduction

Neuropsychiatric disorders such as schizophrenia involve a complex and heterogeneous neurobiology (1). A core feature is the disrupted distinction between the “self” and “non-self,” leading to uncertainty about whether thoughts and actions originate internally or are influenced externally. Such disturbances may contribute to passivity-related symptoms, including auditory verbal hallucinations, thought insertion, and abnormal emotional processing, especially altered responses to emotional stimuli (2). These symptoms, often termed first-rank symptoms, play a critical role in the diagnosis of schizophrenia (3). Prolonged antipsychotic treatment is commonly used during remission. In addition, complementary interventions, such as music therapy and transcranial magnetic stimulation, are applied to improve clinical outcomes (4, 5). Neuroimaging evidence further suggests that these symptoms are closely associated with altered patterns of brain functional connectivity (6–9).

Altered integration of functional connectivity during multi-sensory processing may underlie the neural pathophysiology of self-disorders in schizophrenia (10, 11). Studies combining clinical symptoms and neuroimaging have suggested that these self-disorders are linked to impaired coordination between bottom-up and top-down brain networks (12, 13). For instance, auditory hallucinations are thought to result from a failure of top-down inhibitory control over bottom-up perceptual processing in schizophrenia (14, 15). Moreover, accumulating evidence implicates insular dysfunction in multiple deficits observed in schizophrenia (16). Insula abnormalities may contribute to impaired recognition of emotional facial expressions (17) and difficulties in evaluating or generating emotional vocal expressions (16, 18). Therefore, understanding schizophrenia pathophysiology requires an in-depth assessment of functional interactions both within and between primary sensorimotor and higher-order cognitive networks. To achieve this, functional connectivity analyses based on blood-oxygen-level-dependent (BOLD) signals provide a widely used approach, typically under the assumption of stationary connectivity during scanning (19, 20).

Voxel-mirrored homotopic connectivity (VMHC) is a functional magnetic resonance imaging (fMRI) approach used to assess homotopic functional connectivity between the two hemispheres (21). VMHC directly compares interhemispheric resting-state connectivity by measuring correlations of blood-oxygen-level-dependent (BOLD) time series. This method reflects the communication pattern of information between homologous brain regions and plays a key role in understanding brain information integration. Notably, physiological signals in different frequency bands are generated by distinct functional regions (22). Signals within the same network may also compete or cooperate across different frequency bands (23). However, traditional functional analyses often overlook frequency-specific asymmetry in brain networks. Multi-frequency band VMHC may therefore provide stronger evidence for exploring the neural mechanisms underlying schizophrenia.

Based on previous researches, we hypothesized that the VMHC were frequency dependence in schizophrenic subjects, and these abnormal frequency-specific feature might associated with sensory motor and salience networks.

## 2 Materials and methods

### 2.1 Participants

The participant sample consisted of 39 patients with schizophrenia spectrum disorders (21 female, age = 41.8 ± 9.5) and 38 healthy controls (16 female, age = 40.7 ± 3.2) from a publicly shared dataset - SRPBS Multi-disorder MRI Dataset (https://bicr-resource.atr.jp/srpbsfc/) (24). Patients with schizophrenia were diagnosed on the basis of the Structured Clinical Interview for DSM-IV Axis I Disorders-Patient Edition. Subject demographics are displayed in **Table 1**.

**Table 1:** Participant fundamental information.

Demographic variables	SZ	HC	p-value
Gender (female/male)	21/18	16/22	0.303^a^
Age (year)	41.87 (9.57)	40.71 (3.26)	0.477^b^
FD score	0.18 (0.09)	0.17 (0.06)	0.507^b^
Duration (year)	14.69 (9.56)	–	–
PANSS_P	12.92 (5.66)	–	–
PANSS_N	14.97 (6.07)	–	–
PANSS_G	27.87 (8.65)	–	–
PANSS_T	55.77 (18.58)	–	–

Indicated values are shown as mean (standard deviation).

a, the p-value was calculated by chi-square test.

b, the p-value was calculated by independent samples t-test.

SZ, schizophrenia; HC, healthy controls; FD, framewise displacement; PANSS_P, positive symptom score; PANSS_N, negative symptom score; PANSS_G, general symptom score; PANSS_T, total score.

### 2.2 Data preprocessing

fMRI data was preprocessed according to a standardized preprocessing protocol on Data processing Assistant for Resting Resting-State fMRI (DPARSF). The detailed preprocessing steps were as follows: discard the first 10 volumes, slice-timing correction, realignment, coregistration, normalization and nuisance regression. Nuisance signals including the Friston-24 head motion parameters, white matter, cerebrospinal fluid. Participants with mean framewise displacement (FD) larger than 0.2 were excluded. Filtering with a typical temporal bandpass, including slow-5 bandpass (0.01–0.027 Hz), slow-4 (0.027–0.073 Hz) respectively.

### 2.3 Multi-frequency band voxel-mirrored homotopic connectivity calculation

**Figure 1:**
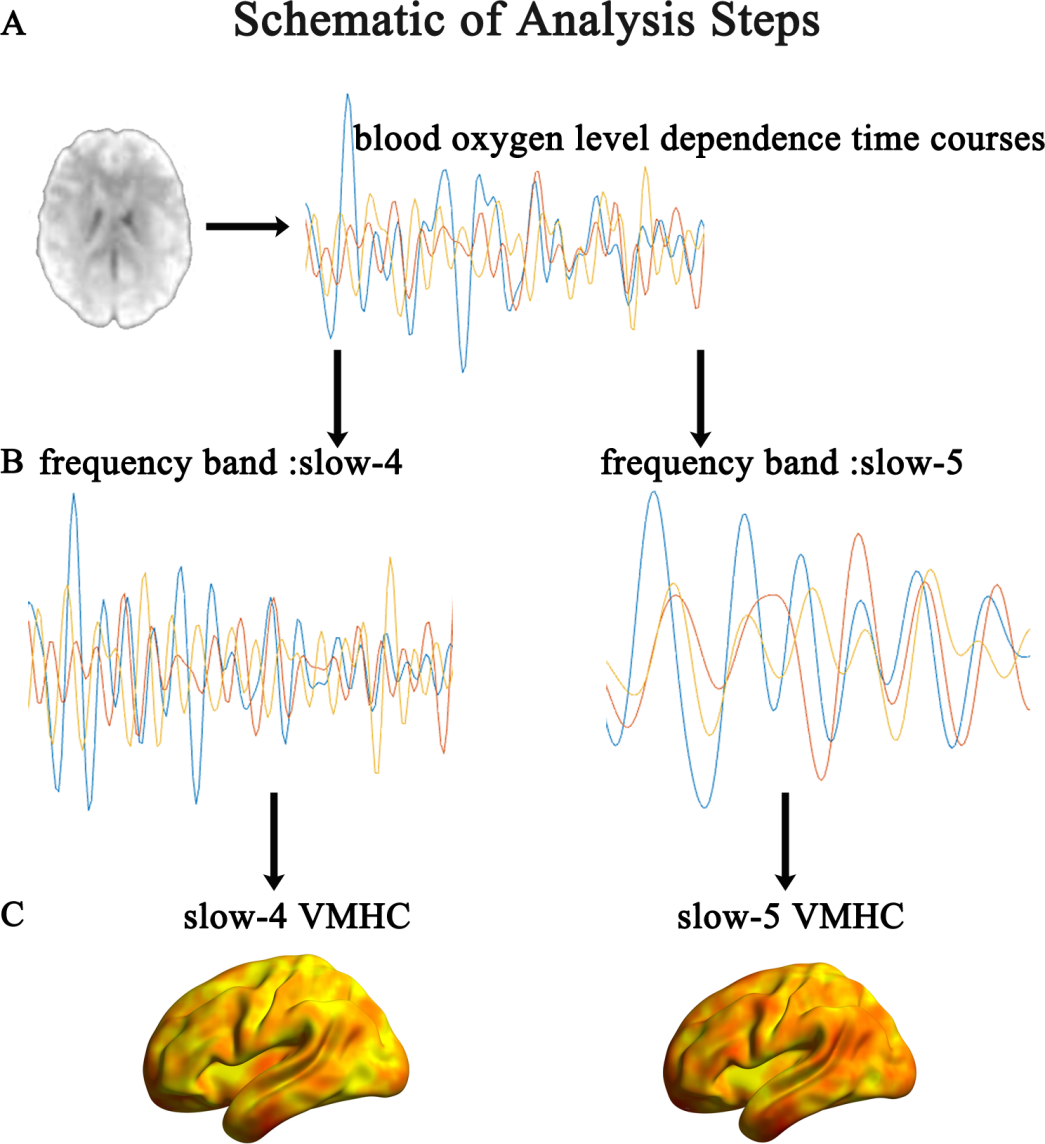
Illustration of analysis steps of VMHC. **(A)** The preprocessed full-length fMRI- BOLD time series of voxels with in gray matter; **(B)** The frequency-specific oscillation of BOLD time series; **(C)** The VMHC maps within slow-4 and slow-5 frequency bands.

Multi-frequency band VMHC analysis was performed using DPARSF software (**Figure 1**). First, we extracted the specifically frequency-band time series of each voxel, including slow-4 and slow-5 in one hemisphere. Second, the Pearson correlation was performed between these time series in one hemisphere and those in the symmetrical hemisphere. Subsequently, the correlation coefficients were transformed into Z-values using Fisher’s Z-transformation. Finally, these maps were spatially smoothed using a Gaussian kernel with a full width at half maximum of 6 mm.

### 2.4 Statistical analysis

After tests for normality, homogeneity of variance and Mauchly’s test of sphericity through Matlab 2020b software, the repeated measures ANCOVA and *post-hoc* analyses were performed to assess the group ∗frequency interaction effect. Age, gender and FD scores were used as the potential confounding covariates in the statistical analysis. The significance threshold of group differences for the ANCOVA was set to uncorrected *p* < 0.001.

### 2.5 Correlations with clinical symptom scores

To examine the relationship between clinical symptoms and altered interaction effects in patients with schizophrenia, we extracted the mean VMHC values from the peak voxel and its surrounding 26 voxels for each significant interaction cluster in the slow-5 and slow-4 frequency bands, respectively. Partial correlation analyses were then performed between PANSS scores and the mean altered frequency-specific VMHC values in patients with schizophrenia, with age, sex, and mean FD as covariates.

## 3 Results

### 3.1 The main effects of group and frequency on VMHC in schizophrenic patients

The spatial distributions of the whole brain mean VMHC were similar in the slow-5 and slow-4 frequency band (**Figure 1C**). Several regions showed VMHC values that were significantly higher than the global mean. These regions were primarily located in key intrinsic connectivity hubs, particularly within the default mode network.

**Figure 2:**
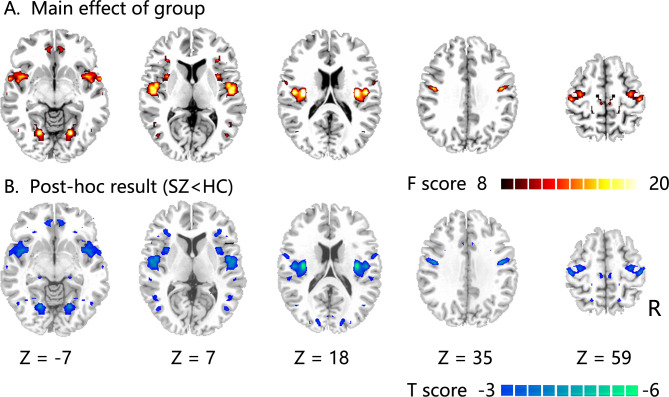
The main effects of group. **(A)** The main effect of group across frequency band on VMHC. **(B)** The *post-hoc* result, blue cold color represents lower VMHC in schizophrenia (SZ) group than in healthy controls (HC).

Based on the repeated-measures ANCOVA, significant main effects of group were observed in several brain regions. Compared with healthy controls, patients with schizophrenia showed decreased VMHC in the superior temporal gyrus, insula, rolandic operculum, Heschl’s gyrus, and middle temporal gyrus (**Table 2**; **Figure 2B**).

**Table 2:** Significant main effects of group and frequency on VMHC through repeated measured ANCOVA.

Brain region	MNI coordinate (x, y, z)	Cluster voxels	Peak F score	The results of Pos-hoc analysis ^a^	
				SZ group vs. HC group	Slow4- VMHC vs. slow5-VMHC
Main effect of group					
Right superior temporal gyrus	36, -15, 15	67	28.29	*t* = -5.42, *p* = 6.95 * 10^-7^	–
Right insula		52		–	–
Right rolandic operculum		33		–	–
Right heschl gyrus		25		–	–
Right temporal pole: superior temporal gyrus		13		–	–
Right Superior temporal gyrus	63,-18,-3	12	25.69		–
Right Middle temporal gyrus		9		–	–
Main effect of frequency				–	
Right precuneus	12, -51, 18	62	20.19	–	*t =* -6.31, *p* = 1.69 * 10^-8^
Right caudate nucleus	6,21,3	48	17.65	–	*t =* -5.42, *p* = 6.77 * 10^-7^
Right lingual gyrus	18, -45, 3	34	16.84	–	*t =* -3.97, *p* = 1.62 * 10^-4^

a, the p-value was calculated by independent samples t-test.

**Figure 3:**
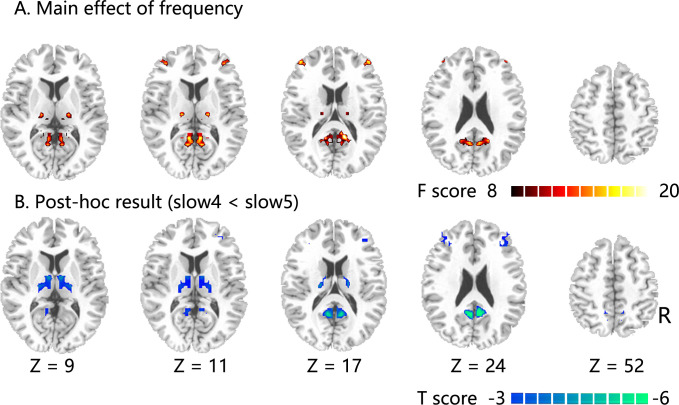
The main effects of frequency. **(A)** The main effect of frequency across frequency group on VMHC. **(B) **The *post-hoc* result, blue cold color represents lower VMHC-slow4 in schizophrenia (SZ) group than VMHC-slow5 in healthy controls (HC).

Significant main effects of frequency were also identified. Compared with the slow-5 frequency band, the slow-4 frequency band showed decreased VMHC in the precuneus and lingual gyrus (**Table 2**; **Figure 3B**).

### 3.2 The group*frequency interaction effect on VMHC in schizophrenic patients

**Figure 4:**
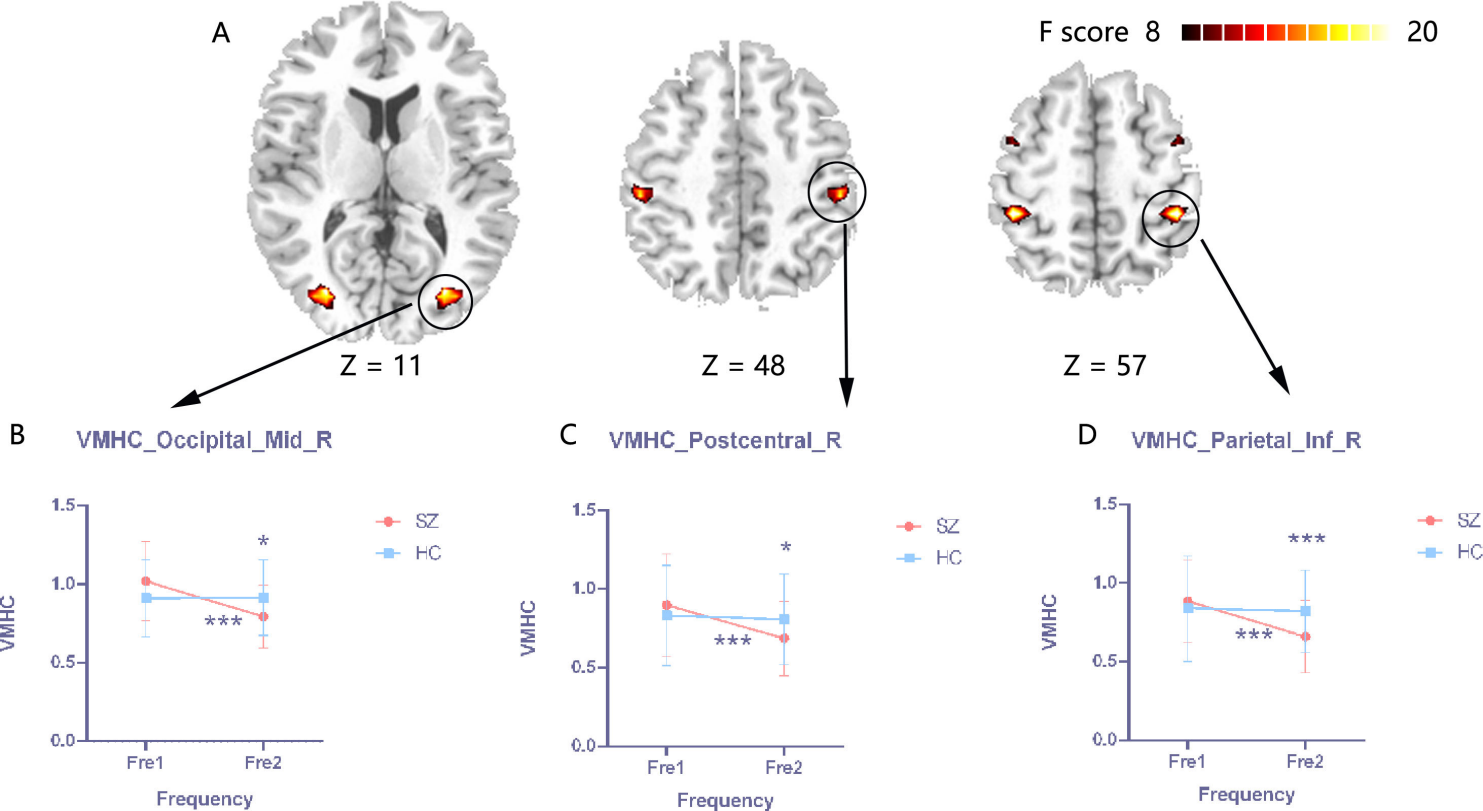
**(A) **The interaction effect between group and frequency on VMHC. **(B)** The VMHC differences (between group and between frequency band) of interaction effects. The data are expressed as the mean value ± standard error. *p < 0.05, ***p < 0.001.

Furthermore, group*frequency interaction on VMHC was observed in inferior parietal gyrus, postcentral gyrus and middle occipital gyrus (**Figure 4A**). *Post-hoc* analysis revealed that the VMHC-slow5 did not show any differences in these region between SZ and HC groups, but VMHC-slow4 was found to be decreased in SZ group (**Figure 4B**; **Table 3**).

**Table 3:** Significant group*frequency interaction on VMHC through repeated measured ANCOVA.

Brain region	MNI coordinate (x, y, z)	Cluster voxels	Peak F score	The results (*t* score and *p* score) of Pos-hoc analysis ^a^			
				slow5-SZ vs. slow5-HC	slow4-SZ vs. slow4-HC	slow5-SZ vs. slow4-SZ	slow5-HC vs. slow4-HC
Inferior parietal gyrus	46, -56, 26	62	21.34	*t* = 0.361 *p* = 0.719	*t* = -3.498 *p* = 8.1 ×10^-3^	*t* = 4.509 *p* = 2.43×10^-5^	*t* = 0.275 *p =* 0.783
Postcentral gyrus	57, -18, 39	29	18.28	*t = 0.718* *p* = 0.475	*t* = -2.547 *p* = 0.012	*t* = 3.572 *p* = 6.3×10^-3^	*t* = 0.374 *p* = 0.709
Middle occipital gyrus	30, -78, 9	26	20.36	*t* = 1.733 *p* = 0.087	*t* = -2.192 *p* = 0.032	*t* = 4.401 *p* = 3.61×10^-5^	*t* = -0.058 *p* = 0.953

a, the p-value was calculated by independent samples t-test.

### 3.3 The relationship between ALFF and clinical symptom score

No significant associations were found between PANSS scores and altered VMHC values in either the slow-5 or slow-4 frequency band among patients with schizophrenia.

## 4 Discussion

Consistent with our hypothesis, the present study revealed significant reductions in VMHC within key sensory networks in schizophrenia, including the primary visual gyrus and sensorimotor regions, suggesting a predominantly diminished interhemispheric synchronization pattern. Moreover, these alterations were frequency-specific, significant group-by-frequency interaction effects were detected in the middle occipital gyrus and postcentral gyrus, and *post-hoc* analyses confirmed reduced slow-4 VMHC in patients relative to controls. Resonating with recent theory and studies (9, 25, 26), these findings highlight the disrupted sensory VMHC in schizophrenia and further provide the critical role of altered functional integration of primary processes, helping understand the pathophysiology of schizophrenia.

Deficits of sensorimotor processing and multisensory functional connectivity integration, first investigated by Kraepelin and Bleuler (27), were well documented as possible pathophysiological mechanisms in schizophrenic subjects (10). Furthermore, the increased time-varying connectivity of sensory and perceptual regions may result in spreading the disrupted internal and external sensory information to the distant high-order regions in schizophrenia (28, 29). In the present study, the lingual gyrus, temporal gyrus, and primary sensorimotor cortex all exhibited reduced VMHC in schizophrenic patients compared to healthy controls. Altered connectivity within and between sensory networks may impair the fidelity of incoming perceptual information and its integration into coherent representations (11, 30). Combining our novel findings and previous studies, these decreased sensory-VMHC may lead to reduced primary perceptual functional foundation, and also might contribute to the deficits of sensory-high order functional connectivity in schizophrenia (31).

Furthermore, the frequency-specific analysis revealed additional insights. In cortical regions, VMHC values were higher at the slow-5 frequency band than at slow-4. Conversely, subcortical regions showed lower VMHC at slow-5 than at slow-4 (32–34). This dissociation suggests that distinct oscillatory mechanisms govern cortical versus subcortical interhemispheric coupling. The significant group-by-frequency interaction effects in the middle occipital gyrus and postcentral gyrus further support a frequency-dependent modulation of interhemispheric connectivity. *Post-hoc* analyses showed that patients had lower VMHC than controls at slow-4, indicating that low-frequency oscillatory synchrony in visual and somatosensory cortices is particularly affected in schizophrenic subjects.

The middle occipital gyrus, located within the dorsal visual pathway (“where” pathway), is essential for spatial processing and visually guided actions (35–38). The postcentral gyrus plays a central role in somatosensory integration (39, 40). Abnormalities in this region have been linked to impaired visual information processing in schizophrenia (41). The postcentral gyrus is the primary somatosensory cortex and plays an important role in sensory integration (42). Reduced VMHC in these dorsal pathway regions may disrupt the transmission of spatial and motion-related visual information, leading to abnormal representations of visual input in schizophrenia (43, 44).

In addition, these abnormalities may be related to cognitive impairment in schizophrenia. Visual and somatosensory signals provide basic sensory input for higher-order cognitive processes. Reduced interhemispheric coordination in the middle occipital gyrus and postcentral gyrus may weaken the reliability of bottom-up sensory transmission (45). This may further affect working memory, reality monitoring, self-representation, and executive control (46, 47). Recent evidence also suggests that cognitive impairment in schizophrenia is associated with distributed functional brain abnormalities, with working memory showing a high level of network integration (44). Therefore, the altered VMHC observed in the present study may reflect impaired sensory-to-cognitive integration in schizophrenia. Future studies should combine VMHC analysis with cognitive assessments to clarify the relationship between frequency-specific brain abnormalities and cognitive deficits.

From a methodological perspective, the present findings underscore the value of examining frequency-specific functional connectivity in patients with schizophrenia. Conventional connectivity analyses typically average across broad frequency ranges, which may mask subtle but clinically relevant patterns. By analyzing distinct frequency bands, we identified differential alterations in cortical and subcortical regions and their interactions, offering a more precise characterization of network dysfunction. This frequency-resolved approach may help reconcile inconsistencies in previous studies and inform more targeted investigations.

Although the present study provides new evidence for understanding the pathophysiological mechanisms of schizophrenia from the perspective of frequency-dependent VMHC alterations, several limitations should be acknowledged. First, this study used publicly available datasets. However, detailed information on medication dosage and treatment duration was not available. Therefore, we could not fully exclude the potential effects of antipsychotic medication on the observed findings. Second, although we identified brain regions showing significant group-by-frequency interaction effects, these alterations were not significantly correlated with PANSS scores in patients with schizophrenia. This may be partly related to the limited sample size, insufficient statistical power, or the heterogeneity of clinical symptoms. Third, the present study was based on a cross-sectional design. Therefore, the current results cannot determine whether the observed abnormalities directly reflect the pathophysiological mechanisms of schizophrenia or dynamic changes during disease progression. Longitudinal studies and intervention-based studies are needed to clarify the clinical and mechanistic significance of frequency-dependent VMHC alterations. Finally, schizophrenia is a highly heterogeneous disorder. The present findings may represent only one aspect of altered interhemispheric coordination. Future studies should integrate multimodal neuroimaging, clinical symptoms, cognitive performance, and treatment response data to further explore potential imaging biomarkers and biological subtypes of schizophrenia (48, 49).

## 5 Conclusions

In conclusion, the study demonstrates that schizophrenia is associated with reduced interhemispheric connectivity in sensory networks, with alterations that are frequency-specific. Disruption of slow-4 oscillatory synchrony in visual and somatosensory cortices may impair multisensory integration and contribute to self-disturbance. These findings offer new perspectives on the pathophysiology of schizophrenia and underscore the value of frequency-resolved analyses in understanding brain network dysfunction.

## Data Availability

The original contributions presented in the study are included in the article/supplementary material. Further inquiries can be directed to the corresponding author.
